# Acetaldehyde-driven mRNA methylation and expression changes in ethanol-metabolizing enzyme genes

**DOI:** 10.1080/15592294.2025.2493865

**Published:** 2025-04-19

**Authors:** Ji Sun Koo, Qiansheng Zhan, Huiping Zhang

**Affiliations:** a Department of Psychiatry, Boston University Chobanian & Avedisian School of Medicine, Boston, MA, USA; b The Biomedical Genetics Section, Department of Medicine, Boston University Chobanian & Avedisian School of Medicine, Boston, MA, USA

**Keywords:** Global m6A RNA methylation, mRNA stop codon region m6A modifications, alcohol-metabolizing enzyme genes, chronic intermittent acetaldehyde exposure, neuroblastoma cell line SH-SY5Y, adenocarcinoma cell line SW620, MazF-RT-qPCR

## Abstract

This study examines how the alcohol metabolite acetaldehyde modulates mRNA methylation and expression of ethanol-metabolizing genes, uncovering its epigenetic role in ethanol metabolism. Using neuron-like (SH-SY5Y) and non-neuronal (SW620) cellular models, we examined the effects of chronic intermittent acetaldehyde (CIA) exposure and subsequent withdrawal (CIA+WD) on global RNA m6A modifications and the methylation and expression of three brain ethanol-metabolizing genes: *CAT* (catalase), *CYP2E1* (cytochrome P450 2E1), and *ALDH2* (aldehyde dehydrogenase 2). A 3-week CIA exposure, with or without 24-hour withdrawal, did not significantly alter global m6A methylation levels in either cell line. However, acetaldehyde exposure/withdrawal induced hypermethylation at the mRNA stop codon regions of *ALDH2* (CIA: *p* = 0.002; CIA+WD: *p* = 0.055) and *CAT* (CIA: *p* = 0.077; CIA+WD: *p* = 0.036) in SH-SY5Y cells, but not in SW620 cells. Furthermore, *ALDH2* mRNA expression was significantly upregulated in both cell types following exposure (SH-SY5Y: *p* = 0.073 [CIA] and 0.00002 [CIA+WD]; SW620: *p* = 0.0009 [CIA] and 0.00008 [CIA+WD]). In contrast, *CYP2E1* mRNA methylation and the expression of *CYP2E1* and *CAT* remained unchanged. These findings highlight the cell-specific epigenetic effects of acetaldehyde, particularly its role in modulating mRNA methylation and expression of *ALDH2*, a key enzyme in alcohol metabolism.

## Introduction

Alcohol use disorder (AUD) is a psychiatric condition characterized by a persistent inability to control alcohol consumption, even when it leads to adverse social, occupational, or health-related consequences. The alcohol flush reaction, or facial flushing, commonly occurs in individuals sensitive to alcohol, particularly those with high alcohol dehydrogenase (ADH) activity (which converts ethanol into the toxic metabolite acetaldehyde) and/or low aldehyde dehydrogenase (ALDH) activity (which metabolizes acetaldehyde into acetate), leading to acetaldehyde accumulation. Excessive alcohol consumption can further elevate acetaldehyde levels, intensifying these flush responses. At higher concentrations, acetaldehyde exerts sedative effects and impairs motor coordination and memory, whereas lower concentrations produce behavioral effects, such as stimulation and reinforcement, akin to those of addictive substances [1]. High concentrations of acetaldehyde also cause symptoms such as headache, dizziness, lightheadedness, and even fainting.

Twin, adoption, family, and linkage studies indicate that approximately half of the risk for developing AUD is attributable to genetic factors [2,3]. Genome-wide association studies (GWAS) have consistently identified variations in alcohol-metabolizing enzyme genes as linked to an increased risk of AUD [4]. For instance, variations in or near the aldehyde dehydrogenase 2 gene (*ALDH2*), which encodes the enzyme ALDH2 primarily responsible for metabolizing the toxic alcohol metabolite acetaldehyde, have been associated with AUD across multiple GWAS [5–7]. These findings suggest that genetic variations affecting the expression of alcohol-metabolizing enzyme genes, such as *ALDH2*, may contribute to increased AUD risk. Beyond genetic predisposition, environmental factors, including parental or sibling alcohol use [8], early adverse life events [9], stress [10], and chronic alcohol consumption, can increase AUD vulnerability.

Environmental factors, including exposure to toxic acetaldehyde from excessive alcohol consumption, are thought to influence AUD risk through epigenetic modifications that alter the expression of alcohol-metabolizing enzyme genes and reward-related genes in specific brain regions. These epigenetic changes can occur at the transcriptional level (e.g., DNA methylation, histone modification, and noncoding RNA regulation of gene expression) or at the post-transcriptional level (e.g., RNA methylation). To date, numerous studies have investigated transcriptional-level epigenetic changes associated with AUD [11]. However, few have examined post-transcriptional RNA methylation changes related to AUD. N6-methyladenosine (m6A) is the most abundant internal modification in eukaryotic RNAs, playing a crucial role in regulating RNA translation, stability, and splicing [12–14]. M6A is particularly enriched in 3’ untranslated regions (3’ UTRs) and near stop codons [15]. M6A methylation near the stop codon can affect translation termination, ribosome recycling, mRNA stability, and overall translation efficiency [16]. Our recent study found that chronic ethanol exposure affected global m6A RNA methylation levels, as well as m6A modifications around the mRNA stop codon of three opioid receptor genes (*OPRM1*, *OPRD1*, and *OPRK1*) [17]. Given the dual properties of acetaldehyde as both a toxic and reinforcing alcohol metabolite, we propose that acetaldehyde may also influence global m6 RNA methylation levels, as well as m6A RNA methylation in key alcohol metabolism-related genes and AUD-relevant genes.

**Figure 1.**
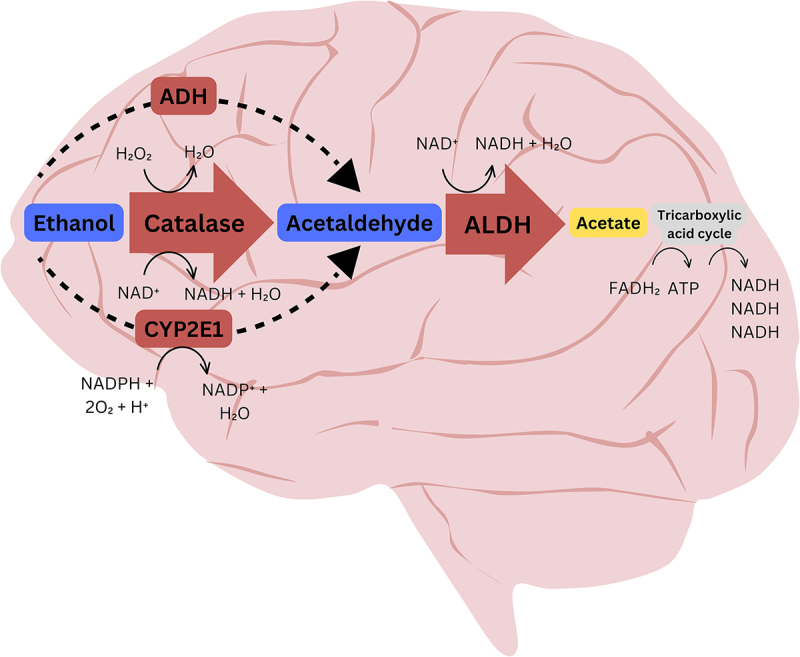
Alcohol metabolizing enzymes and alcohol metabolism in the brain. Alcohol in the brain is mainly metabolized to acetaldehyde by catalase, and acetaldehyde is converted to acetate by aldehyde dehydrogenases (ALDH).

In this study, we investigated global m6A RNA methylation changes induced by acetaldehyde exposure, as well as alterations in m6A RNA methylation within the stop codon region of three key genes [the catalase gene (*CAT*), the cytochrome P450 2E1 gene (*CYP2E1*), and the aldehyde dehydrogenase 2 gene (*ALDH2*)] involved in alcohol metabolism in the brain. The primary enzymes involved in alcohol metabolism are alcohol dehydrogenase (ADH) family members, aldehyde dehydrogenase 2 (ALDH2), cytochrome P450 (CYP2E1), and catalase [18]. In the liver, alcohol is mainly metabolized to acetaldehyde by ADH, with involvement from cytochrome CYP2E1 and the enzyme catalase. Moreover, the high activity of liver ALDH2 rapidly converts acetaldehyde to acetate, ensuring that little acetaldehyde enters the bloodstream. However, ADH, the primary ethanol-metabolizing enzyme for converting alcohol to acetaldehyde, is not physiologically active in the brain. Evidence suggests that the brain can produce acetaldehyde through local ethanol metabolism, primarily involving catalase and CYP2E1 (Figure 1) [19]. Catalase is estimated to metabolize around 60–70% of ethanol in the brain, whereas CYP2E1 is responsible for generating less than 10–20% of acetaldehyde [20]. Brain-generated acetaldehyde is converted into non-toxic acetate by ALDH2, a specific isozyme of the ALDH family found in higher concentrations in the liver, brain, and heart [21].

Thus, ALDH2 is a key enzyme responsible for eliminating acetaldehyde, a toxic byproduct of alcohol metabolism. Interestingly, acetaldehyde has been shown to induce the production of ALDH2 [22]. Additionally, studies indicate that acetaldehyde exposure impacts epigenetic regulation by influencing DNA methylation and histone modifications at specific genomic loci [22]. Based on these findings, we hypothesize that acetaldehyde may also modulate the expression of genes (particularly those encoding alcohol-metabolizing enzymes) by altering RNA methylation patterns at the posttranscriptional level. To test this hypothesis, we utilized neuronal and non-neuronal cell models to evaluate the effects of acetaldehyde exposure on global m6A RNA methylation and m6A modifications near the mRNA stop codons of three alcohol metabolism-related genes: *CAT*, *CYP2E1*, and *ALDH2*. M6A methylation levels in these genes were quantified using MazF digestion followed by reverse transcription and quantitative PCR (MazF-RT-qPCR), a method designed to measure m6A methylation at m6ACA motifs [17]. Overall, this study demonstrates that acetaldehyde exposure increases mRNA methylation and/or expression levels of key alcohol-metabolizing enzyme genes, particularly *ALDH2*. These findings enhance our understanding of the epitranscriptomic mechanisms underlying AUD and may inform the development of novel therapies for AUD by targeting the epigenetic or expression status of alcohol-metabolizing enzyme genes.

## Materials and methods

### SH-SY5Y cells and SW620

The human neuroblastoma cell line SH-SY5Y is widely utilized in experimental toxicology and neurobiological research due to its expression of a greater number of brain-specific genes compared to other cell types, making it an ideal model for this study. The non-neuronal cell line SW620, derived from colorectal adenocarcinoma, is commonly used in cancer and toxicology research. Both cell lines were purchased from the American Type Culture Collection (ATCC; Manassas, VA, USA) and cultured in a 1:1 mixture of EMEM (ATCC) and F-12K medium (ATCC) supplemented with 1% penicillin-streptomycin (Corning, Manassas, VA, USA) and 10% (vol/vol) fetal bovine serum (Corning, Woodland, CA, USA). Cultures were maintained at 37°C in a humidified atmosphere of 5% CO₂.

### Chronic intermittent acetaldehyde (CIA) exposure and withdrawal (WD)

**Figure 2.**
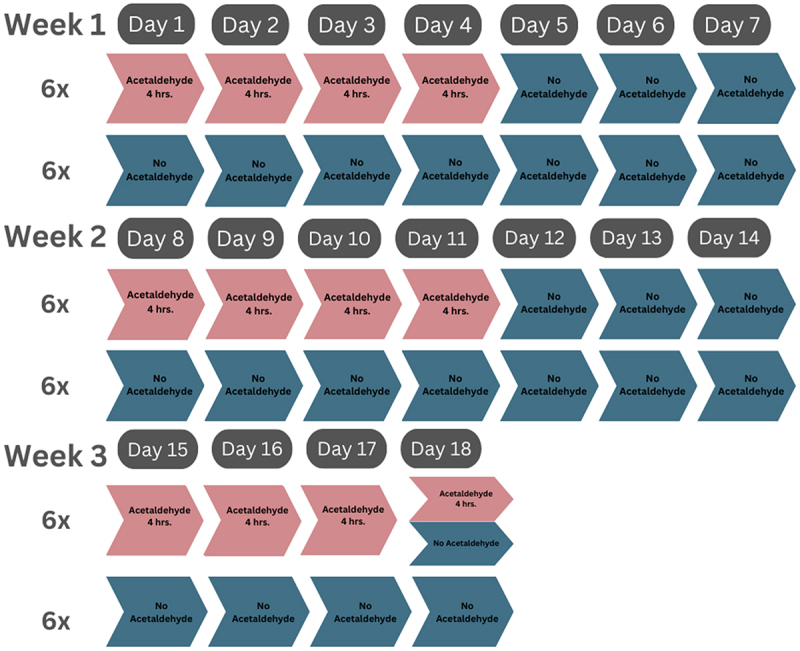
A 3-week chronic intermittent acetaldehyde (CIA) exposure followed by a 24-hr withdrawal (WD). The figure illustrates the study design, which includes three groups of SH-SY5Y and SW620 cells, respectively: (1) control cells (without acetaldehyde treatment); (2) cells with chronic intermittent acetaldehyde (CIA) exposure; and (3) cell with CIA exposure followed by a 24-hour withdrawal (WD).

In our previous study, we exposed SH-SY5Y and SW620 cells to chronic intermittent ethanol (CIE) for 3 weeks [17]. In the current experiment, we applied the same treatment protocol using acetaldehyde instead of ethanol. As shown in Figure 2, each cell type was seeded at a density of about 5 × 10^5^ cells/dish into 12 culture dishes (60 × 15 mm; Corning, Corning, NY, USA) (2 cell types × 12 dishes = 24 dishes in total). After 24 hours, six dishes of each cell line were cultured in medium containing 30 μM acetaldehyde, while the remaining six dishes of each cell line were cultured in acetaldehyde-free medium. After four hours, the medium was replaced with fresh acetaldehyde-free medium for all 24 dishes (Day 1). This treatment protocol was repeated daily from Day 2 to Day 4. From Day 5 to Day 7, all cells were cultured exclusively in acetaldehyde-free medium. This weekly exposure cycle was repeated for the second (Days 8–14) and the first three days of the third week (Days 15–17). On Day 17, cells from both lines were harvested after the 4-hour acetaldehyde exposure and divided into two groups. Each group consisted of six dishes of SH-SY5Y cells and six dishes of SW620 cells. One group was cultured for an additional 24 hours without acetaldehyde to model a withdrawal period, while the other group was harvested immediately. This resulted in three conditions for each cell line: chronic intermittent acetaldehyde (CIA)-treated cells, CIA-treated cells followed by a 24-hour withdrawal period (CIA+WD), and untreated control cells. In total, 18 collections (6 + 6 + 6 = 18 dishes) of each cell line (2 × 18 = 36 total) were acquired and stored at −80°C (6 dishes per condition per cell line).

The acetaldehyde concentration of 30 μM was selected based on published reports of peripheral blood acetaldehyde levels in alcoholic subjects. Korsten et al. reported a mean blood acetaldehyde plateau of 42.7 ± 1.2 μM [23], while Eriksson et al. observed blood acetaldehyde concentrations of 23 μM [24]. Similarly, Lindros et al. demonstrated elevated blood acetaldehyde levels ranging from 10–50 μM after ethanol ingestion [25]. Based on these findings, 30 μM was chosen as a physiologically relevant concentration for treating SH-SY5Y and SW620 cells.

Prior to the formal experiment, a dose-response study was conducted using both neuron-like SH-SY5Y cells and non-neuronal SW620 cells. The cells were cultured for 7 days in medium containing 0, 30, 100, 500, or 1,000 µM of acetaldehyde, with a daily 4-hour exposure. The viability of SH-SY5Y cells remained largely unaffected at concentrations up to 500 µM, but at 1,000 µM, a noticeable decrease in viability was observed under the microscope. In contrast, SW620 cells demonstrated greater resistance to acetaldehyde, showing no significant changes in viability even at 1,000 µM. Please refer to Supplementary Figure S1.

### Total RNA extraction

Total RNA was extracted from SH-SY5Y and SW620 cells using QIAGEN miRNeasy Mini Spin Columns (QIAGEN, Germantown, MD, USA). Residual DNA in RNA samples was removed by DNase I digestion directly on the silica membrane of the RNeasy spin column, utilizing the QIAGEN RNase-Free DNase Set (QIAGEN, Germantown, MD, USA). The purity and concentration of the extracted RNA were assessed with a NanoDrop 1000 Spectrophotometer (Thermo Fisher Scientific, Waltham, MA, USA).

### Global m6A RNA methylation assay

The global m6A RNA methylation levels of all 36 total RNA samples were measured using the m6A RNA Methylation Quantification Kit (Colorimetric) (Abcam, Waltham, MA, USA), following the protocol outlined in our previous study [17]. This kit utilizes an enzyme-linked immunosorbent assay (ELISA)-like method to quantify global RNA methylation levels. Briefly, about 200 ng of total RNA was coated onto the flat bottom of 8-well strips. Capture antibody, detection antibody, and enhancer solutions were sequentially added to each well according to the manufacturer’s instructions. Developer and stop solutions were subsequently added, and the absorbance at 450 nm was measured using a SpectraMax® i3 Multi-Mode Detection Platform (Molecular Devices, San Jose, CA, USA). A standard curve was generated using a series of known m6A amounts per well *versus* OD450nm, enabling the quantification of global m6A RNA methylation levels for each of the 36 total RNA samples.

The global m6A mRNA methylation levels of total RNA samples extracted from SH-SY5Y and SW620 cells were calculated as described in our previous study [17]. A standard curve and a non-linear regression equation were generated using ELISAcalc software (BioTNT, Shanghai, China) to determine the relationship between the m6A amount (ng) in positive controls and OD_450 nm_. Among the nonlinear curve models generated by ELISAcalc, the 4-parameter logistic (4PL) curve best modeled the relationship between m6A amounts (ng) and OD_450 nm_, and a non-linear regression equation [y = (A – D)/[1 + (x/C)^B] + D; y: m6A amount (ng), x: OD_450 nm_; A = 0; B = 1.832782; C = 0.063092; D = 1.628543] was obtained (Supplementary Figure S2). The m6A amount (ng) in each total RNA sample was calculated according to this equation using the corresponding OD_450 nm_ value [OD_450 nm_ (RNA samples) – OD_450 nm_ (Negative Control)]. The percentage of m6A in a total RNA sample was calculated using the formula: m6A% = [m6A amount (ng)/S (ng)] × 100% [S: amount (ng) of input RNA].

### CAT, CYP2E1, and ALDH2 mRNA stop codon region m6A methylation assay

The m6A RNA methylation levels near the mRNA stop codons of three alcohol-metabolizing enzyme genes (*CAT*, *CYP2E1*, and *ALDH2*) were quantified using MazF digestion followed by reverse transcription and quantitative polymerase chain reaction (MazF-RT-qPCR). First, approximately 500 ng of total RNA was digested with 20 units of mRNA interferase-MazF enzyme (Takara Bio, Ann Arbor, MI, USA) at 37°C for 30 minutes in a 10 μl reaction mixture containing 1× MazF Buffer and 20 units of RiboLock RNase Inhibitor (Thermo Fisher Scientific, Cambridge, MA, USA). The MazF-treated RNA was then reverse transcribed into cDNA using the RevertAid Reverse Transcription Kit (Thermo Fisher Scientific, Cambridge, MA, USA) in a 20 μl reaction containing 200 units of RevertAid reverse transcriptase, 20 units of RiboLock RNase Inhibitor, 1 mm dNTPs, 5 μM random hexamer primers, and 1× Reaction Buffer. Finally, quantitative polymerase chain reactions (qPCR) were performed using the synthesized cDNA as a template to assess the m6A methylation levels near the mRNA stop codon regions of the three alcohol-metabolizing enzyme genes (*CAT*, *CYP2E1*, and *ALDH2*).

**Figure 3.**
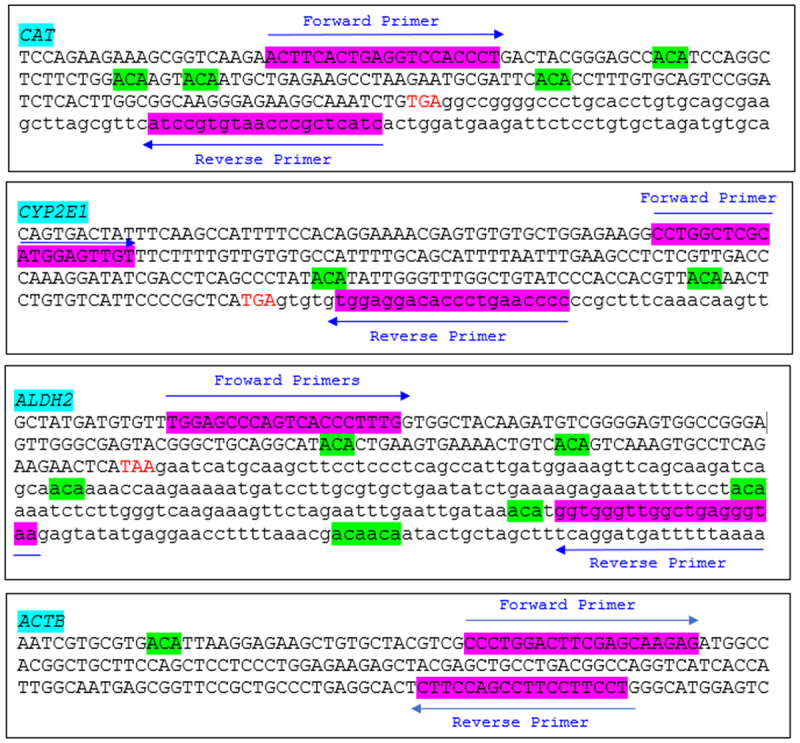
Forward and reverse primers designed for amplifying cDNA sequences around mRNA stop codons of three alcohol-metabolizing enzyme genes and the reference gene *ACTB*. *CAT*: the catalase gene; *CYP2E1*: the cytochrome P450 2E1 gene; *ALDH2*: the aldehyde dehydrogenase gene; *ACTB*: the beta-actin gene. Forward and reverse primers are highlighted in magenta, while m6ACA motifs are marked in green. TGA or TAA represents the stop codon of the mRNA.

NCBI Primer Blast (www.ncbi.nlm.nih.gov/tools/primer-blast/) was used to design primers pairs targeting the mRNA stop codon regions of three alcohol-metabolizing enzyme genes. As shown in Figure 3, the catalase gene (*CAT*) primer pair (CAT_F: 5’ ACTTCACTGAGGTCCACCCT 3;’ CAT_R: 5’ ATCCGTGTAACCCGCTCATC 3’) amplifies a 202-bp sequence containing four ACA motifs. The cytochrome P450 2E1 gene (*CYP2E1*) primer pair (CYP2E1_F: 5’ CCTGGCTCGCATGGAGTTGT 3;’ CYP2E1_R: 5’ TGGAGGACACCCTGAACCCC 3’) spans a 185-bp sequence with two ACA motifs. The aldehyde dehydrogenase 2 gene (*ALDH2*) primer pair (ALDH2_F: 5’ TGGAGCCCAGTCACCCTTTG 3;’ ALDH2_R: 5’ GGTGGGTTGGCTGAGGGTAA 3’) amplifies a 309-bp sequence containing seven ACA motifs. Additionally, a primer pair targeting the β-actin gene (*ACTB*), used as a housekeeping reference gene, was designed. The *ACTB* primer pair (ACTB_F: 5’-CCCTGGACTTCGAGCAAGAG-3;’ ACTB_R: 5’-CCAGGAAGGAAGGCTGGAAG-3’) amplifies a 144-bp region of the *ACTB* coding sequence, which does not contain any ACA motifs. The PCR products for the three alcohol-metabolizing enzyme genes and the reference gene (*ACTB*) were visualized *via* agarose gel electrophoresis (Supplementary Figure 3).

Using the SYBR Green PCR Master Mix (Thermo Fisher Scientific, Waltham, MA, USA) and the QuantStudio 12K Flex system, qPCR was performed to quantify the relative m6A methylation levels near the mRNA stop codon regions of three alcohol-metabolizing enzyme genes (*CAT*, and *CYP2E1*, and *ALDH2*), with the β-actin gene (ACTB) serving as a reference. The cycling protocol began with an initial denaturation at 95°C for 10 minutes, followed by 40 cycles of two steps: denaturation at 95°C for 15 seconds and annealing/extension at 60°C for 1 minute. After qPCR, a melting curve analysis was conducted to confirm amplification specificity. The threshold cycle (Ct) values for the three alcohol-metabolizing enzyme genes and the reference gene (*ACTB*) were analyzed using the QuantStudio™ 12K Flex Software. The relative mRNA methylation levels (ΔCt) of the three alcohol-metabolizing enzyme genes were normalized to that of the reference gene *ACTB* [ΔCt = Ct(*CAT*, *CYP2E1*, or *ALDH2*) – Ct(*ACTB*)]. Since MazF specifically cleaves mRNAs at ACA sequences but not methylated ACA (or m6ACA) sequences, a higher ΔCt value indicates a lower m6A methylation level, whereas a lower ΔCt value reflects a higher m6A methylation level.

### CAT, CYP2E1, and ALDH2 mRNA expression assay

RT-qPCR was used to assess the mRNA expression levels of the three alcohol-metabolizing enzyme genes (*CAT, CYP2E1, and ALDH2*), with the β-actin gene (*ACTB*) serving as the reference gene. The procedure was identical to the steps outlined above for measuring relative m6A methylation levels, except that MazF digestion of total RNA was omitted. Instead, cDNA was directly synthesized from the extracted total RNA samples. The Ct value of *ACTB* was used as a reference to normalize the relative mRNA expression levels of the three alcohol-metabolizing enzyme genes. The relative mRNA expression levels (ΔCt) of three alcohol-metabolizing enzyme genes were normalized to that of the reference gene *ACTB* [ΔCt = Ct (*CAT*, *CYP2E1*, or *ALDH2*) – Ct (*ACTB*)]. A higher ΔCt value corresponds a lower mRNA expression level, while a lower ΔCt value indicates a higher mRNA expression level.

### Statistical analyses

A one-way analysis of variance (ANOVA) was performed to evaluate statistically significant differences in global and gene-specific RNA methylation, as well as RNA expression levels, among three groups of cells: acetaldehyde-untreated (Control), chronic intermittent acetaldehyde (CIA) exposure, and CIA exposure followed by a 24-hour acetaldehyde withdrawal (CIA+WD). Tukey’s honestly significant difference (HSD) test was used for pairwise comparisons of RNA methylation and expression levels between the groups (Control, CIA, and CIA+WD).

## Results

### Acetaldehyde exposure or withdrawal did not significantly affect global m6A RNA methylation levels

Global m6A RNA methylation changes were assessed following a 3-week chronic intermittent acetaldehyde (CIA) exposure or a 3-week CIA exposure with a subsequent 24-hr withdrawal (CIA + WD) in both neuron-like SH-SY5Y and non-neuronal SW620 cells. One-way ANOVA revealed no significant differences among the three experimental groups (Control, CIA, and CIA+WD) in either cell line [SH-SY5Y: F(2,15) = 0.928, *p* = 0.417; SW620: F(2,15) = 1.15, *p* = 0.344]. Post hoc Tukey HSD pairwise comparisons further confirmed that neither CIA nor CIA+WD significantly affected global m6A RNA methylation levels in either cell type (Supplementary Figure 4).

### ALDH2 mRNA methylation and expression changes induced by acetaldehyde exposure or subsequent withdrawal

**Figure 4.**
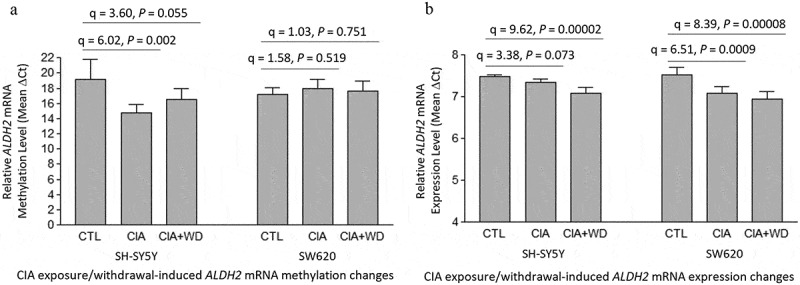
Chronic intermittent acetaldehyde (CIA) exposure/withdrawal-induced mRNA methylation and expression changes in the aldehyde dehydrogenase 2 gene (*ALDH2*). CTL: Control SH-SY5Y or SW620 cells (without acetaldehyde exposure). CIA: a 3-week chronic intermittent acetaldehyde (CIA) exposure. CIA+WD: a 3-week CIA exposure followed by a 24-hr acetaldehyde withdrawal.

One-way ANOVA revealed a significant difference in methylation levels around the stop codon region of *ALDH2* mRNA among the three treatment groups (CTL, CIA, and CIA+WD) in neuron-like SH-SY5Y cells [F(2,15) = 9.18, *p* = 0.002]. Post hoc Tukey HSD pairwise comparisons confirmed that both CIA and CIA+WD treatments significantly induced hypermethylation around the stop codon region of *ALDH2* mRNA compared to the control group [CIA *vs*. CTL: q = 6.02, *p* = 0.002; CIA+WD *vs*. CTL: q = 3.60, *p* = 0.055] (Figure 4(a)). However, neither acetaldehyde exposure nor withdrawal significantly influenced overall methylation levels in this region in non-neuronal SW620 cells, as determined by one-way ANOVA [F(2,15) = 0.61, *p* = 0.557] and post hoc Tukey HSD pairwise comparisons (Figure 4(a)).

Additionally, one-way ANOVA demonstrated that acetaldehyde exposure or withdrawal caused significant differences in *ALDH2* mRNA expression levels among the three treatment groups in both cell types [SH-SY5Y: F(2,15) = 23.82, *p* = 0.00002; SW620: F(2,15) = 19.38, *p* = 0.00007]. Post hoc Tukey HSD pairwise comparisons indicated that both CIA and CIA+WD treatments elevated *ALDH2* mRNA expression levels in neuron-like SH-SY5Y cells (CIA *vs*. CTL: q = 3.38, *p* = 0.073; CIA+WD *vs*. CTL: q = 9.62, *p* = 0.00002) and in non-neuronal SW620 cell (CIA *vs*. CTL: q = 6.51, *p* = 0.0009; CIA+WD *vs*. CTL: q = 8.39, *p* = 0.00008) (Figure 4(b)).

### CAT mRNA methylation and expression changes induced by acetaldehyde exposure or withdrawal

**Figure 5.**
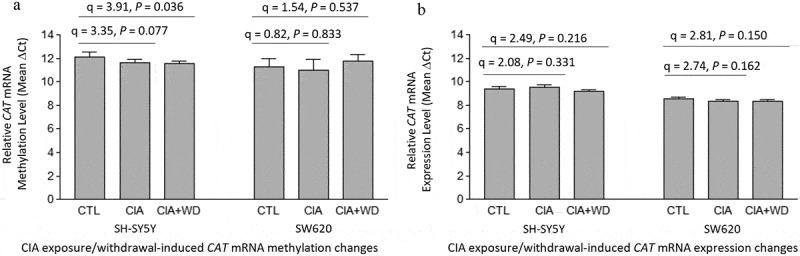
Chronic intermittent acetaldehyde (CIA) exposure/withdrawal-induced mRNA methylation and expression changes in the catalase gene (*CAT*). CTL:Control SH-SY5Y or SW620 cells (without acetaldehyde exposure). CIA:a 3-week chronic intermittent acetaldehyde (CIA) exposure. CIA+WD:a 3-week CIA exposure followed by a 24-hr acetaldehyde withdrawal.

One-way ANOVA revealed a significant effect of acetaldehyde exposure or withdrawal on *CAT* mRNA methylation levels in neuron-like SH-SY5Y cells [F(2,15) = 4.47, *p* = 0.030]. Post hoc Tukey HSD pairwise comparisons indicated a trend toward hypermethylation around the stop codon region of *CAT* mRNA in acetaldehyde-exposed SH-SY5Y cells (CIA *vs*. CTL: q = 3.35, *p* = 0.077). Additionally, methylation levels in this region were significantly higher in CIA+WD-treated SH-SY5Y cells compared to control SH-SY5Y cells (CIA+WD *vs*. CTL: q = 3.91, *p* = 0.036) (Figure 5(a)). In contrast, no significant effect of CIA or CIA+WD treatments on *CAT* mRNA methylation was observed in non-neuronal SW620 cells, as indicated by one-way ANOVA [F(2,15) = 1.43, *p* = 0.270] or post hoc Tukey HSD pairwise comparisons (Figure 5(a)).

Although one-way ANOVA showed a significant difference in *CAT* mRNA expression levels among the three treatment groups (CTL, CIA, and CIA+WD) of neuron-like SH-SH5Y cells [F(2,15) = 5.24, *p* = 0.019], post hoc Tukey HSD pairwise comparisons did not identify a significant effect of CIA or CIA+WD treatments on *CAT* mRNA expression in SH-SY5Y cells [CIA *vs*. CTL: q = 2.08, *p* = 0.331; CIA+WD *vs*. CTL: q = 2.49, *p* = 0.216] (Figure 5(b)). Similarly, no significant changes in *CAT* mRNA expression levels were observed in SW620 cells treated with CIA or CIA+WD, as shown by one-way ANOVA [F(2, 15) = 2.57, *p* = 0.110] and post hoc Tukey HSD pairwise comparisons (Figure 5(b)).

### No significant changes in CYP2E1 mRNA methylation or expression were detected following acetaldehyde exposure or withdrawal

Acetaldehyde exposure or withdrawal did not significantly alter *CYP2E1* mRNA methylation in either cell type, as determined by one-way ANOVA [SH-SY5Y: F(2,15) = 2.68, *p* = 0.103; SW620: F(2,15) = 2.36, *p* = 0.129] and post hoc Tukey HSD pairwise comparisons (Supplementary Figure 5a). Similarly, *CYP2E1* mRNA expression remained unaffected in both cell types under the same conditions, as indicated by one-way ANOVA [SH-SY5Y: F(2,15) = 0.71, *p* = 0.509; SW620: F(2,15) = 3.39, *p* = 0.061] and post hoc Tukey HSD pairwise comparisons (Supplementary Figure 5b).

## Discussion

Acetaldehyde, a highly reactive and toxic metabolite of ethanol, exerts diverse biological effects, including tissue damage, carcinogenesis, oxidative stress, the alcohol flush reaction, and a significant role in addiction. Genetic factors that affect the enzymes involved in alcohol metabolism play a crucial role in acetaldehyde accumulation. Increased activity of enzymes (such as ADH, catalase, and CYP2E1) responsible for converting ethanol to acetaldehyde or reduced activity of ALDH2 which metabolizes acetaldehyde to acetate, can lead to toxic acetaldehyde buildup and flushing responses, particularly in East Asian populations. Heavy alcohol consumption also contributes to acetaldehyde accumulation. Interestingly, acetaldehyde levels remain low even after binge drinking. While the blood ethanol concentration in binge drinkers is approximately 40 mm [26–28], blood acetaldehyde levels are typically only around 10–50 µM [23–25]. The mechanisms underlying the rapid clearance of acetaldehyde after alcohol consumption remain poorly understood.

We hypothesize that acetaldehyde modulates the expression of alcohol-metabolizing enzyme genes through feedback mechanisms, reducing the activity of ADH, catalase, and CYP2E1 while enhancing ALDH2 activity *via* epigenetic mechanisms, particularly RNA methylation. To investigate this hypothesis, we employed both neuronal and non-neuronal cell models to explore acetaldehyde-induced changes in mRNA methylation and expression of these genes. Given the minimal ADH activity in the brain, our study focused on three key genes: the catalase gene (*CAT*), the cytochrome P450 2E1 gene (*CYP2E1*), and the aldehyde dehydrogenase 2 gene (*ALDH2*). RNA methylation modifications may regulate the expression of alcohol metabolizing enzymes at the post-transcriptional (RNA) level, potentially accelerating the clearance of toxic acetaldehyde and preventing its accumulation in the body after alcohol consumption. Our study results provide partial support for this hypothesis.

Among the three alcohol-metabolizing enzyme genes, *ALDH2* is the most significantly affected in terms of mRNA methylation and expression following acetaldehyde exposure or withdrawal. This highlights the critical role of ALDH2 in converting toxic acetaldehyde into non-toxic acetate. Interestingly, acetaldehyde exerts a greater impact on *ALDH2* mRNA methylation in neuron-like SH-SY5Y cells compared to non-neuronal SW620 cells (Figure 4(a)), likely reflecting the heightened sensitivity of brain neuronal cells to acetaldehyde toxicity relative to peripheral tissues or cells. Additionally, the expression of *ALDH2* was upregulated in both cell types (Figure 4(b)). In SH-SY5Y cells, the hypermethylation of *ALDH2* mRNA correlated with its increased expression following acetaldehyde exposure, suggesting an epitranscriptomic mechanism driving this upregulation. However, the precise mechanism underlying *ALDH2* mRNA upregulation in SW620 cells exposed to acetaldehyde remains unclear. It is possible that acetaldehyde exposure induces other types of epigenetic modifications, such as DNA methylation or histone modifications, which may contribute to the elevated expression of *ALDH2* mRNA in SW620 cells.

*CAT*, which encodes the catalase enzyme involved in converting ethanol to acetaldehyde in the brain, exhibits relatively minor changes in mRNA methylation and expression following acetaldehyde exposure or withdrawal, compared to *ALDH2*. Acetaldehyde-induced hypermethylation of *CAT* mRNA, similar to that observed for *ALDH2* mRNA, was detected exclusively in neuron-like SH-SY5Y cells but not in non-neuronal SW620 cells (Figure 5(a)). Furthermore, no significant changes in *CAT* mRNA expression levels were observed in either cell type following acetaldehyde exposure (Figure 5(b)). This finding was unexpected, as we had hypothesized that acetaldehyde exposure would reduce *CAT* expression, thereby limiting the production of toxic acetaldehyde from consumed alcohol. Our results suggest that *CAT* plays a less critical role than *ALDH2* in regulating acetaldehyde levels in the body after alcohol consumption. Consequently, *CAT* appears to be less sensitive to low levels of acetaldehyde exposure in terms of mRNA methylation and expression compared to *ALDH2*.

Moreover, we did not observe a significant effect of acetaldehyde on the mRNA methylation or expression of *CYP2E1* (Supplementary Figure S5). Like catalase, CYP2E1 is a key enzyme involved in converting alcohol to acetaldehyde in the brain, linking alcohol consumption to oxidative damage and neurological dysfunction. Studies have shown that at low to moderate ethanol levels, alcohol metabolism primarily depends on ADH and catalase in both neuronal and non-neuronal cells. At high ethanol concentrations, however, CYP2E1 activity increases due to its induction by chronic or excessive alcohol exposure, particularly when ADH and catalase activities are saturated [29]. It is likely that exposure to low levels of acetaldehyde (30 µM, equivalent to the blood acetaldehyde concentration after binge drinking) is insufficient to induce epigenetic modifications that would alter *CYP2E1* expression.

Additionally, while our previous study reported significant changes in global RNA methylation levels in ethanol-exposed SH-SY5Y and SW620 cells [17], similar alterations were not observed in these cell types following acetaldehyde exposure. These findings underscore the complexity of environmental toxicants’ effects on RNA methylation, which may not consistently manifest as global changes. The specific outcomes likely depend on factors such as the nature of the toxicant, the type of RNA affected, and the cellular context. Furthermore, acetaldehyde-induced hypermethylation and hypomethylation of different types of RNA may counterbalance each other, potentially resulting in no significant net changes in overall RNA methylation levels.

The present study had several key limitations. First, we focused exclusively on acetaldehyde-induced RNA methylation changes in neuron-like SH-SY5Y cells and non-neuronal peripheral SW620 cells. However, glial cells play a critical role in alcohol metabolism and neuroprotection against alcohol and its metabolite acetaldehyde. Future research should explore acetaldehyde-induced RNA methylation and gene expression changes in glial cells, particularly in genes associated with alcohol metabolism and alcohol use disorder. Second, our analysis was limited to three key enzyme genes (*CAT*, CYP2E1, and *ALDH2*) involved in alcohol metabolism. Other genes in this pathway may also undergo epigenetic modifications and expression changes following acetaldehyde exposure or withdrawal, warranting further investigation. Third, the MazF-qPCR method we used has inherent limitations. MazF cleaves only single-stranded RNAs immediately upstream of unmodified ACA motifs but not methylated m6ACA motifs [30]. To gain a more comprehensive view, future research should explore acetaldehyde-induced RNA methylation at additional m6A sites within DRACH consensus sequences (where D = A, G, or U; *R* = A or G; and H = A, C, or U) [31,32]. Fourth, we did not assess whether acetaldehyde-induced mRNA methylation changes in *ALDH2, CAT*, and *CYP2E1* correlate with their protein expression. Prior studies indicate that ethanol consumption downregulates ALDH2 expression [33], while increasing the expression or activity of catalase [34] and CYP2E1 [35]. In contrast, acetaldehyde exposure has been reported to upregulate ALDH2 expression [22] or activity [36]. Our findings align with these reports, showing that acetaldehyde exposure increases *ALDH2* mRNA expression in both cell types. However, its effects on catalase and CYP2E1 expression remain unclear. To address this, future studies should examine whether acetaldehyde-induced *ALDH2* mRNA methylation changes correspond to protein expression changes using immunoblotting (Western blotting). Fifth, there is a concern regarding the maintenance of acetaldehyde concentration (30 µM) in the culture medium due to its high volatility. However, acetaldehyde has a very high solubility in water (approximately 1,000 g/L or 22.7 M at 20°C), which supports the feasibility of maintaining this concentration. We believe that a 30 µM concentration can be sustained in the culture medium, especially considering that physiological blood levels of acetaldehyde in heavy drinkers range from 20–50 µM. Moreover, the cells were exposed to acetaldehyde at this low concentration for only 4 hours per day. Even if some acetaldehyde is lost due to evaporation, the reduction in concentration is unlikely to be substantial due to its high solubility in aqueous media. Finally, the mechanism by which acetaldehyde increases *ALDH2* and *CAT* mRNA methylation remains unknown. Further research should determine whether acetaldehyde exposure alters the expression of RNA methylation regulators, such as RNA methylases and demethylases.

## Conclusions

In conclusion, the present study demonstrates that acetaldehyde exposure or withdrawal increases mRNA methylation and/or expression levels of key alcohol-metabolizing enzyme genes, particularly *ALDH2*, thereby potentially affecting alcohol metabolism as well as the physiological and behavioral effects of alcohol and its metabolite, acetaldehyde. Among the three alcohol metabolizing enzyme genes (*CAT*, *CYP2E1*, and *ALDH2*) expressed in the brain, *ALDH2* is the most significantly affected by acetaldehyde in terms of mRNA methylation and expression. This finding underscores the critical role of *ALDH2* in detoxifying acetaldehyde, particularly following episodes of binge drinking.

## Supplementary Material

Supplemental Material

## Data Availability

All data generated in this study are freely available within the manuscript and the supplementary materials.

## Abbreviations

CAT: the catalase gene
CYP2E1: the cytochrome P450 family 2 subfamily E member 1 gene
ALDH2: the aldehyde dehydrogenase 2 (ALDH2) gene
CIA: chronic intermittent acetaldehyde exposure
CIA+WD: chronic intermittent acetaldehyde exposure followed by a 24-hr withdrawal
SH-SY5Y: human neuroblastoma cell Line
SW620: human colon carcinoma cell line
RT-qPCR: reverse transcription followed by quantitative polymerase reaction
MazF-RT-qPCR: MazF digestion followed by RT-qPCR
