# Supplementary figures and images for: Acetaldehyde-driven mRNA methylation and expression changes in ethanol-metabolizing enzyme genes
Source: Epigenetics. 2025 Apr 19;20(1):2493865. doi: 10.1080/15592294.2025.2493865 (PMC12013419; doi:10.1080/15592294.2025.2493865)

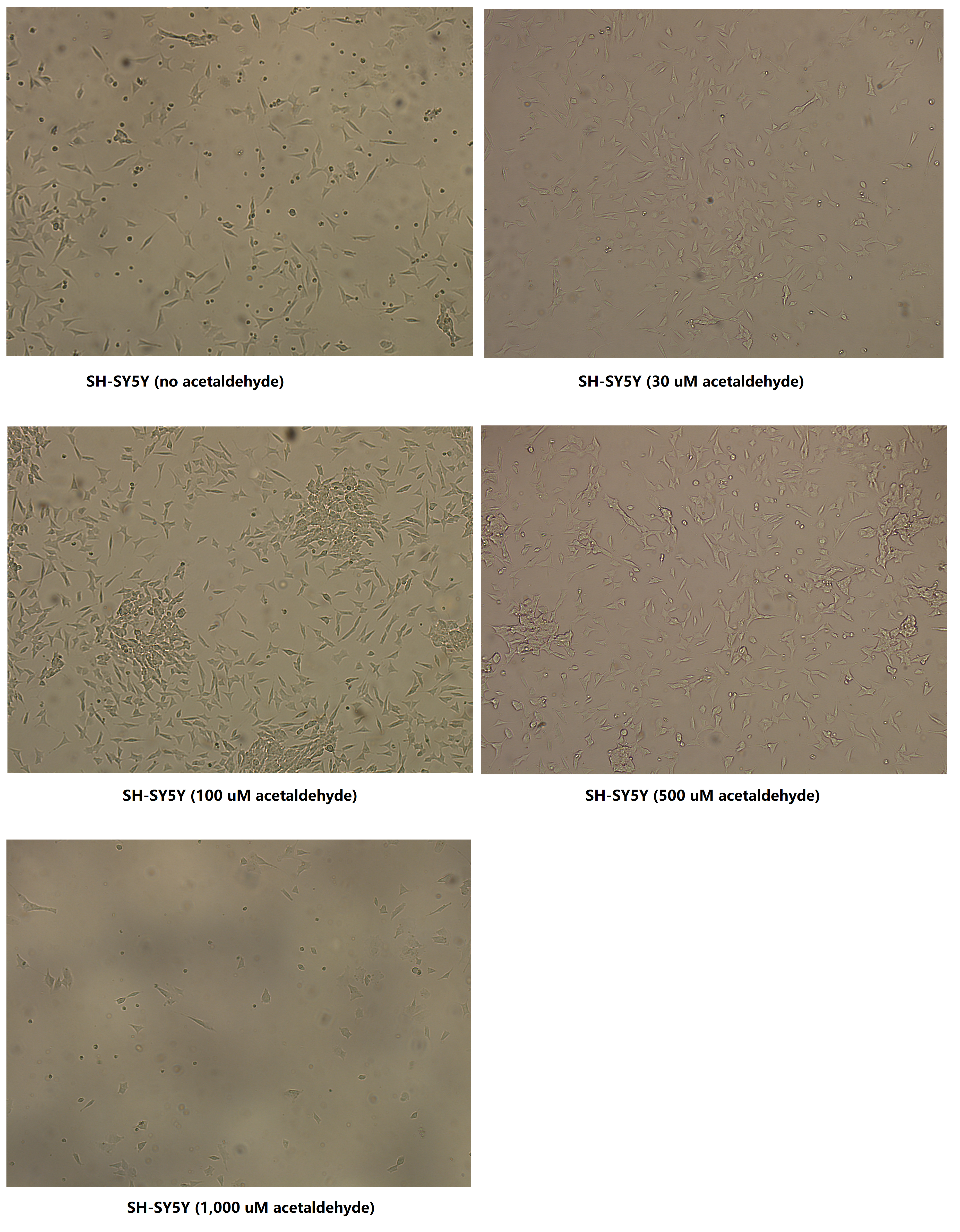

Supplement: Supplemental Material [file KEPI_A_2493865_SM0511.zip › Supplementary information/Figure_S1a_SHSY5Y_exposed_acetaldehyde_dose_effect.tif]

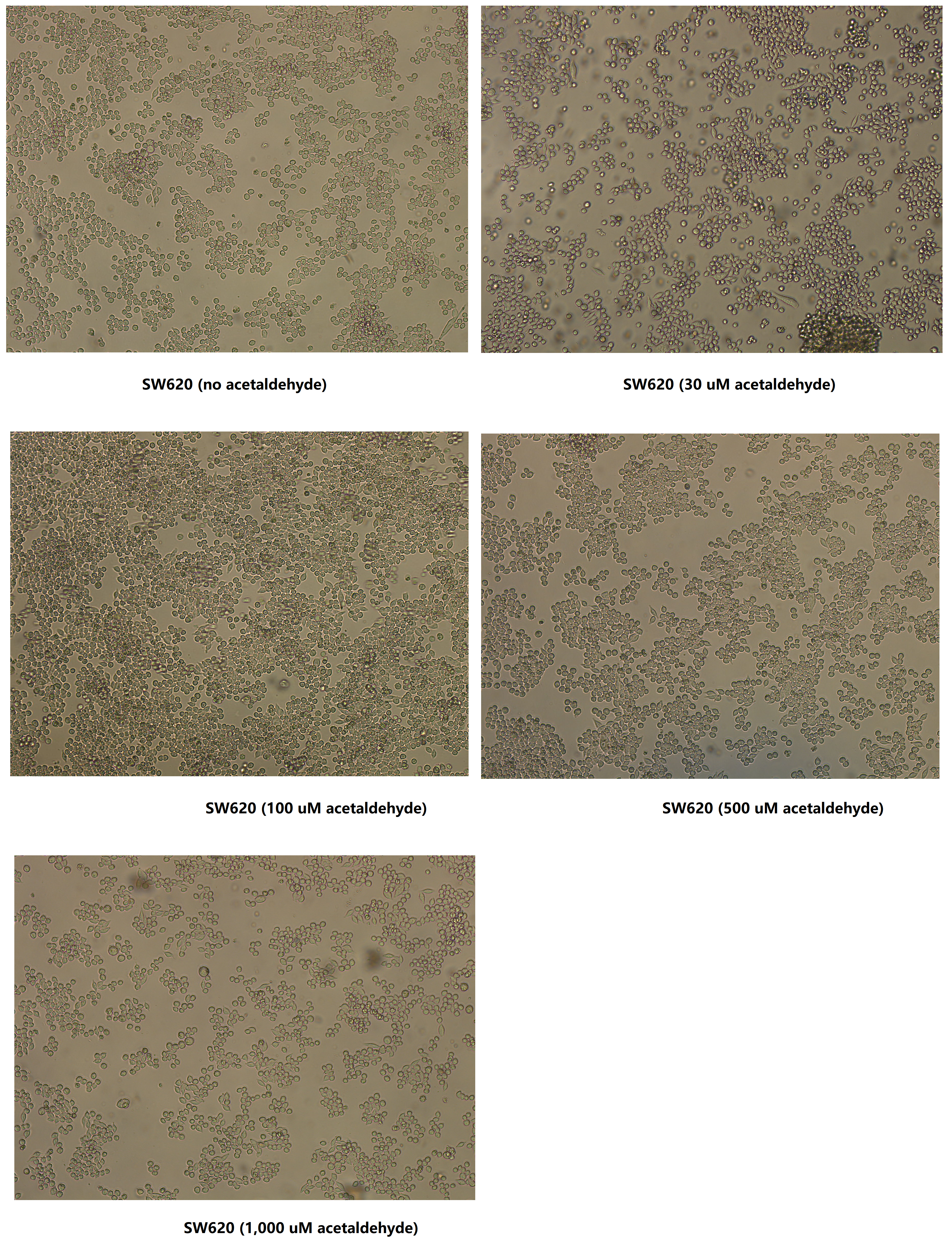

Supplement: Supplemental Material [file KEPI_A_2493865_SM0511.zip › Supplementary information/Figure_S1b_SW620_exposed_acetaldehyde_dose_effect.tif]

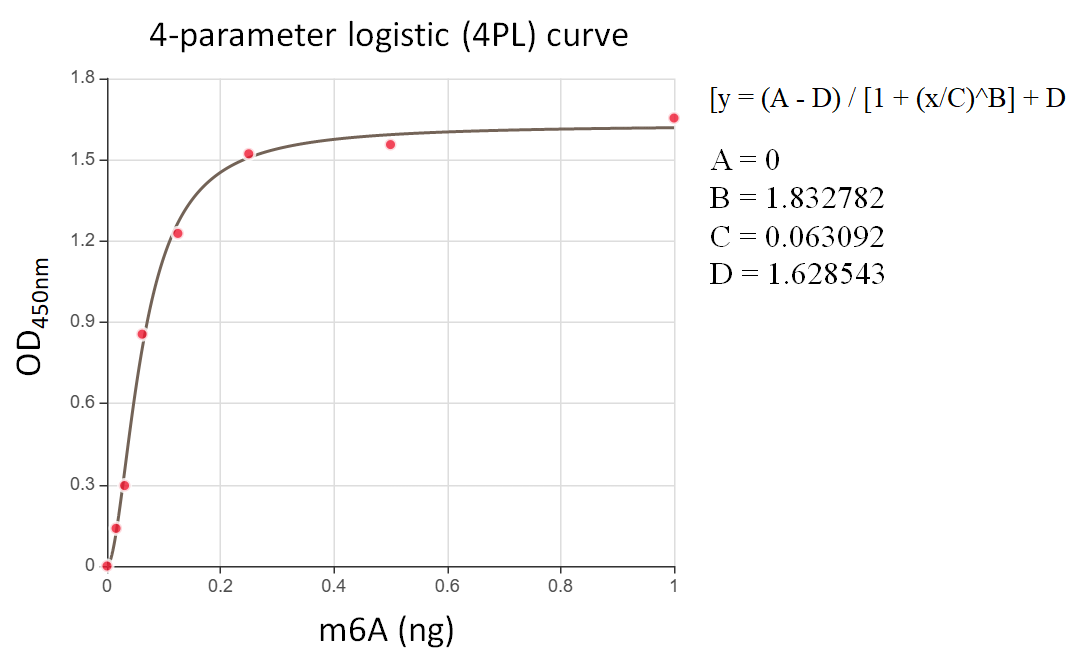

Supplement: Supplemental Material [file KEPI_A_2493865_SM0511.zip › Supplementary information/Figure_S2_Global_RNA_methylation_standard_curve.tif]

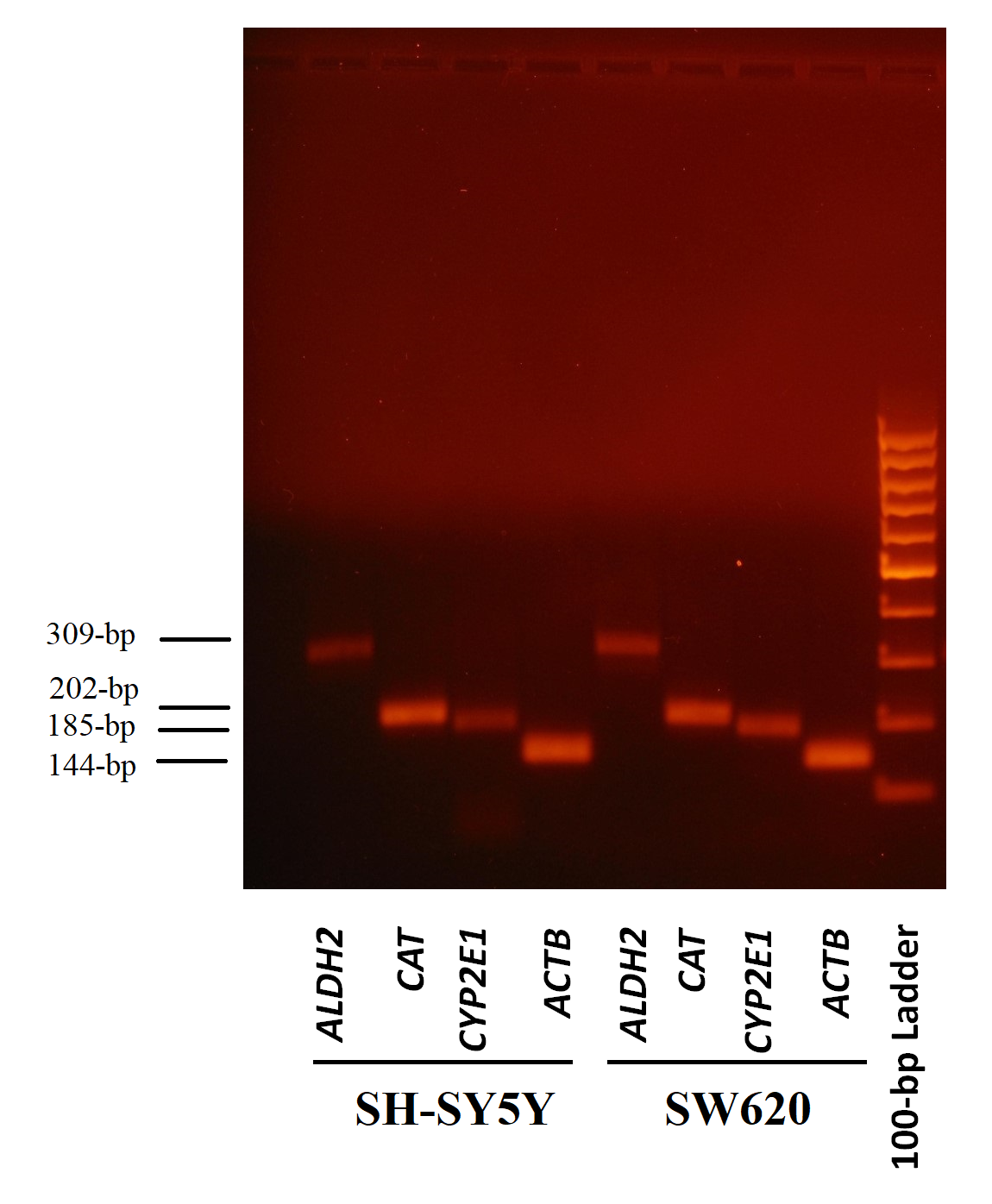

Supplement: Supplemental Material [file KEPI_A_2493865_SM0511.zip › Supplementary information/Figure_S3_Agarose_gel_electrophoresis.tif]

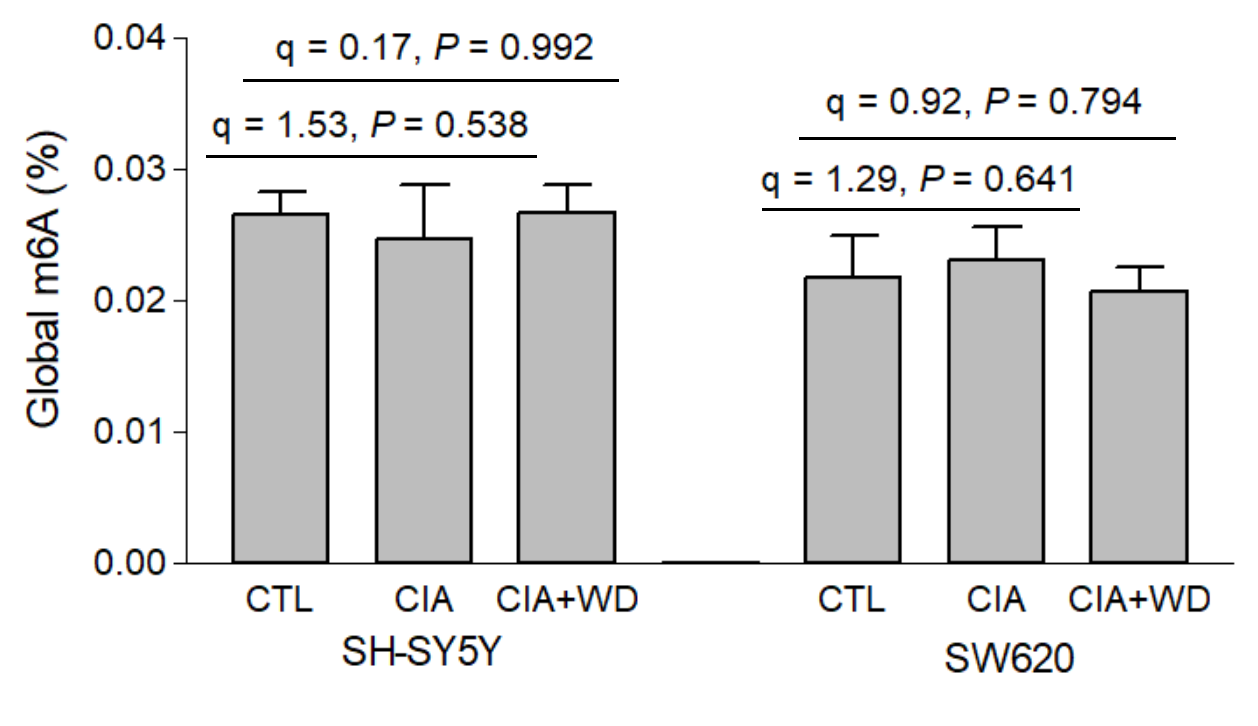

Supplement: Supplemental Material [file KEPI_A_2493865_SM0511.zip › Supplementary information/Figure_S4_CIA_exposure_induced_global_RNA_m6A_methylation_changes.tif]

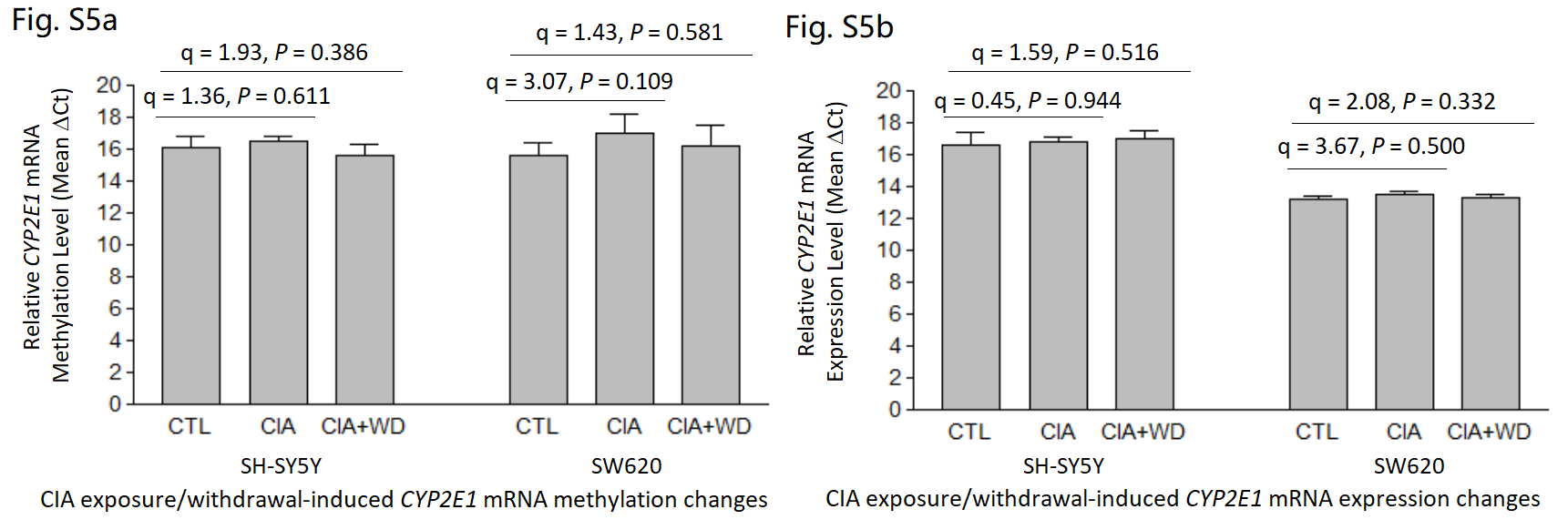

Supplement: Supplemental Material [file KEPI_A_2493865_SM0511.zip › Supplementary information/Figure_S5_CIA_WD_CYP2E1_mRNA_methylation_expression_SHSY5Y_SW620.tif]
